# 2-Bromo-1-(4-methoxy­phen­yl)ethanone

**DOI:** 10.1107/S1600536809033303

**Published:** 2009-08-26

**Authors:** Jian Zhang, Ling-hua Zhuang, Guo-wei Wang

**Affiliations:** a Department of Light Chemical Engineering, College of Food Science and Light Engineering, Nanjing University of Technology, Nanjing 210009, People’s Republic of China

## Abstract

The title compound, C_9_H_9_BrO_2_, prepared by the reaction of 4-methoxy­acetophenone and cupric bromide, , is approximately planar (r.m.s. deviation 0.0008 Å). In the crystal, weak inter­molecular aromatic C—H⋯O_carbon­yl_ hydrogen-bonding inter­actions result in a one-dimensional chain structure.

## Related literature

For background to hydrazone compounds, see: Domiano *et al.* (1984 ▶); Li *et al.* (1988 ▶); Sadik *et al.* (2004 ▶). For background to thia­zole compounds, see: Shinagawa *et al.* (1997 ▶); Shivarama *et al.*(2003 ▶); Dinçer *et al.* (2005 ▶); Zhang *et al.* (2009 ▶). For bond-length data, see: Allen *et al.* (1987 ▶).
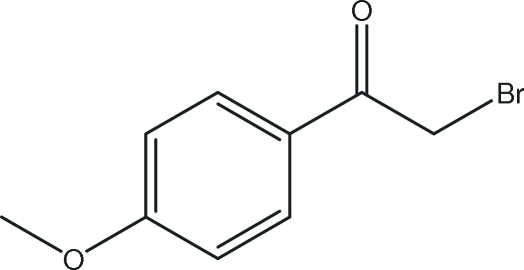


## Experimental

### 

#### Crystal data

C_9_H_9_BrO_2_
*M* *_r_* = 229.06
Monoclinic, 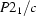

*a* = 7.7360 (15) Å
*b* = 12.441 (3) Å
*c* = 10.048 (2) Å
β = 111.42 (3)°
*V* = 900.3 (4) Å^3^
*Z* = 4
Mo *K*α radiation
μ = 4.52 mm^−1^
*T* = 305 K
0.20 × 0.10 × 0.10 mm

#### Data collection

Enraf–Nonius CAD-4 diffractometer
Absorption correction: ψ scan (North *et al.*, 1968 ▶) *T*_min_ = 0.465, *T*_max_ = 0.661
1634 measured reflections
1634 independent reflections
924 reflections with *I* > 2σ(*I*)
3 standard reflections every 200 reflections intensity decay: 9%

#### Refinement

*R*[*F* ^2^ > 2σ(*F* ^2^)] = 0.054
*wR*(*F* ^2^) = 0.116
*S* = 1.01
1634 reflections
109 parameters
H-atom parameters constrained
Δρ_max_ = 0.35 e Å^−3^
Δρ_min_ = −0.53 e Å^−3^

### 

Data collection: *CAD-4 Software* (Enraf–Nonius, 1989 ▶); cell refinement: *CAD-4 Software*; data reduction: *XCAD4* (Harms & Wocadlo, 1995 ▶); program(s) used to solve structure: *SHELXS97* (Sheldrick, 2008 ▶); program(s) used to refine structure: *SHELXL97* (Sheldrick, 2008 ▶); molecular graphics: *SHELXTL* (Sheldrick, 2008 ▶); software used to prepare material for publication: *SHELXTL*.

## Supplementary Material

Crystal structure: contains datablocks global, I. DOI: 10.1107/S1600536809033303/zs2006sup1.cif

Structure factors: contains datablocks I. DOI: 10.1107/S1600536809033303/zs2006Isup2.hkl

Additional supplementary materials:  crystallographic information; 3D view; checkCIF report

## Figures and Tables

**Table 1:** Hydrogen-bond geometry (Å, °)

*D*—H⋯*A*	*D*—H	H⋯*A*	*D*⋯*A*	*D*—H⋯*A*
C7—H7*A*⋯O1^i^	0.93	2.58	3.505 (7)	171

Symmetry code: (i) 
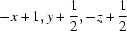
.

**Fig. 1.**
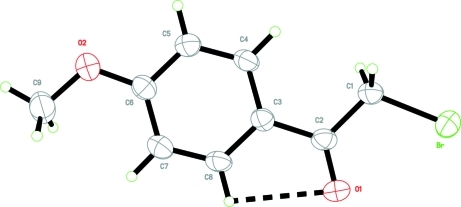
A view of the molecular structure of (I) showing the atom-numbering scheme with non-H atoms drawn as 30% displacement ellipsoids. The intramolecular hydrogen bond is shown as a dashed line.

**Fig. 2.**
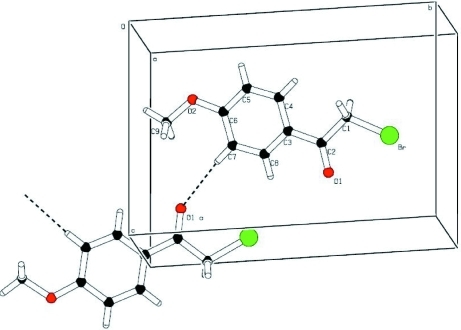
The crystal packing of (I) showing hydrogen bonds as dashed lines.

## supplementary crystallographic information

### Comment

The chemistry of hydrazones, owing to their coordinating capability, pharmacological activity, antibacterial and antifungal properties, and their use in analytical chemistry as highly selective extractants, has been intensively investigated (Domiano *et al.*, 1984; Li *et al.*, 1988; Sadık *et al.*, 2004). In addition, many thiazole compounds are of considerable importance because of their antibacterial and anti-inflammatory activity (Shinagawa *et al.*, 1997; Shivarama *et al.*, 2003; Dinçer *et al.*, 2005). We have focused our synthetic and structural studies on new derivatives of thiazole-substituted hydrazones (Zhang *et al.*, 2009). We report here the crystal structure of a bromo-substituted methoxyacetophenone, the title compound C_9_H_9_BrO_2_ (I), which is a very important intermediate for the synthesis of thiazole-substituted hydrazones.

In (I), all bond lengths are within normal ranges (Allen *et al.*, 1987). The presence of a strong intramolecular aromatic C8–H···O1_carbonyl_ hydrogen bond (Table 1) forms a pseudo five-membered ring [O1/C2/C3/C8/H8A with an r.m.s. deviation 0.0069 Å], maintaining essential coplanarity of the ketone side chain with the benzene ring (Fig. 1) [torsion angle: C1–C2–C3–C8, -178.0 (5)°]. The methoxy group is similarly essentially coplanar [torsion angle: C5–C6–O2–C9, 172.0 (6)°].

The molecules of (I) associate through weak intermolecular aromatic C—H···O_carbonyl_ hydrogen bonds forming one-dimensional chains which extend along the *b* axial direction in the unit cell (Fig.2).

### Experimental

4-Methoxyacetophenone (1.50 g, 0.01 mol) was dissolved in 50 ml ethyl acetate, cupric bromide (3.36 g, 0.015 mol) was added and the mixture was refluxed for *ca.* 3 h. On cooling, the solid which separated was filtered and recrystallized from ethyl acetate. Crystals of (I) suitable for X-ray diffraction were obtained by slow evaporation of ethyl acetate. ^1^H NMR (CDCl_3_, δ, p.p.m.) 8.17 (d, 2 H), 7.49 (d, 2 H), 4.5 (s, 2 H), 3.81 (s,3 H).

### Refinement

All H atoms were positioned geometrically, with C—H = 0.93 Å, and constrained to ride on their parent atoms, with *U*_iso_(H) = *xU*_eq_(C), where *x*= 1.5 for methyl H and *x* = 1.2 for methylene and aromatic H atoms.

### Crystal data

**Table:**

C_9_H_9_BrO_2_	*F*(000) = 456
*M**_r_* = 229.06	*D*_x_ = 1.690 Mg m^−^^3^
Monoclinic, *P*2_1_/*c*	Mo *K*α radiation, λ = 0.71073 Å
Hall symbol: -P 2ybc	Cell parameters from 27 reflections
*a* = 7.7360 (15) Å	θ = 1–25°
*b* = 12.441 (3) Å	µ = 4.52 mm^−^^1^
*c* = 10.048 (2) Å	*T* = 305 K
β = 111.42 (3)°	Block, colorless
*V* = 900.3 (4) Å^3^	0.20 × 0.10 × 0.10 mm
*Z* = 4	

### Data collection

**Table:**

Enraf–Nonius CAD-4 diffractometer	924 reflections with *I* > 2σ(*I*)
Radiation source: fine-focus sealed tube	*R*_int_ = 0.0000
graphite	θ_max_ = 25.3°, θ_min_ = 2.7°
ω/2θ scans	*h* = −9→8
Absorption correction: ψ scan (North *et al.*, 1968)	*k* = 0→14
*T*_min_ = 0.465, *T*_max_ = 0.661	*l* = 0→12
1634 measured reflections	3 standard reflections every 200 reflections
1634 independent reflections	intensity decay: 9%

### Refinement

**Table:**

Refinement on *F*^2^	Primary atom site location: structure-invariant direct methods
Least-squares matrix: full	Secondary atom site location: difference Fourier map
*R*[*F*^2^ > 2σ(*F*^2^)] = 0.054	Hydrogen site location: inferred from neighbouring sites
*wR*(*F*^2^) = 0.116	H-atom parameters constrained
*S* = 1.00	*w* = 1/[σ^2^(*F*_o_^2^) + (0.048*P*)^2^] where *P* = (*F*_o_^2^ + 2*F*_c_^2^)/3
1634 reflections	(Δ/σ)_max_ = 0.001
109 parameters	Δρ_max_ = 0.35 e Å^−^^3^
0 restraints	Δρ_min_ = −0.53 e Å^−^^3^

### Special details

**Table:**

Geometry. All e.s.d.'s (except the e.s.d. in the dihedral angle between two l.s. planes) are estimated using the full covariance matrix. The cell e.s.d.'s are taken into account individually in the estimation of e.s.d.'s in distances, angles and torsion angles; correlations between e.s.d.'s in cell parameters are only used when they are defined by crystal symmetry. An approximate (isotropic) treatment of cell e.s.d.'s is used for estimating e.s.d.'s involving l.s. planes.
Refinement. Refinement of *F*^2^ against ALL reflections. The weighted *R*-factor *wR* and goodness of fit *S* are based on *F*^2^, conventional *R*-factors *R* are based on *F*, with *F* set to zero for negative *F*^2^. The threshold expression of *F*^2^ > σ(*F*^2^) is used only for calculating *R*-factors(gt) *etc*. and is not relevant to the choice of reflections for refinement. *R*-factors based on *F*^2^ are statistically about twice as large as those based on *F*, and *R*- factors based on ALL data will be even larger.

### Fractional atomic coordinates and isotropic or equivalent isotropic displacement parameters (Å^2^)

**Table:**

	*x*	*y*	*z*	*U*_iso_*/*U*_eq_	
Br	0.20597 (10)	0.66539 (6)	0.07859 (7)	0.0717 (3)	
O1	0.3621 (7)	0.8754 (4)	0.2083 (5)	0.0848 (15)	
C1	0.1809 (8)	0.8016 (5)	−0.0164 (6)	0.0576 (17)	
H1A	0.2308	0.7957	−0.0918	0.069*	
H1B	0.0500	0.8190	−0.0608	0.069*	
O2	0.2187 (6)	1.3075 (4)	−0.1487 (5)	0.0689 (13)	
C2	0.2781 (8)	0.8917 (5)	0.0820 (6)	0.0534 (16)	
C3	0.2657 (7)	0.9994 (5)	0.0181 (6)	0.0478 (14)	
C4	0.1597 (8)	1.0221 (5)	−0.1242 (6)	0.0593 (17)	
H4A	0.0957	0.9671	−0.1849	0.071*	
C5	0.1493 (9)	1.1250 (5)	−0.1751 (7)	0.0633 (18)	
H5A	0.0780	1.1390	−0.2703	0.076*	
C6	0.2432 (8)	1.2086 (5)	−0.0870 (7)	0.0538 (16)	
C7	0.3497 (8)	1.1859 (5)	0.0560 (7)	0.0600 (17)	
H7A	0.4138	1.2406	0.1172	0.072*	
C8	0.3586 (8)	1.0831 (5)	0.1051 (6)	0.0564 (16)	
H8A	0.4297	1.0688	0.2003	0.068*	
C9	0.2906 (10)	1.3980 (6)	−0.0610 (8)	0.083 (2)	
H9A	0.2625	1.4618	−0.1186	0.124*	
H9B	0.4227	1.3908	−0.0151	0.124*	
H9C	0.2353	1.4028	0.0102	0.124*	

### Atomic displacement parameters (Å^2^)

**Table:**

	*U*^11^	*U*^22^	*U*^33^	*U*^12^	*U*^13^	*U*^23^
Br	0.0721 (5)	0.0588 (5)	0.0722 (5)	0.0064 (4)	0.0121 (3)	0.0076 (4)
O1	0.104 (4)	0.067 (3)	0.050 (3)	0.002 (3)	−0.011 (3)	0.003 (2)
C1	0.054 (4)	0.060 (4)	0.050 (3)	0.002 (3)	0.008 (3)	−0.003 (3)
O2	0.079 (3)	0.054 (3)	0.070 (3)	−0.007 (2)	0.021 (2)	−0.002 (2)
C2	0.042 (3)	0.060 (4)	0.049 (4)	0.007 (3)	0.006 (3)	−0.005 (3)
C3	0.036 (3)	0.050 (4)	0.053 (4)	−0.001 (3)	0.011 (3)	−0.003 (3)
C4	0.060 (4)	0.056 (4)	0.049 (3)	−0.002 (3)	0.005 (3)	−0.012 (3)
C5	0.067 (4)	0.059 (4)	0.052 (4)	0.005 (4)	0.007 (3)	0.000 (3)
C6	0.047 (4)	0.056 (4)	0.058 (4)	−0.004 (3)	0.018 (3)	−0.007 (3)
C7	0.054 (4)	0.062 (5)	0.058 (4)	−0.011 (3)	0.013 (3)	−0.010 (3)
C8	0.044 (4)	0.064 (4)	0.049 (3)	−0.004 (3)	0.001 (3)	−0.005 (3)
C9	0.087 (5)	0.063 (5)	0.096 (5)	−0.012 (4)	0.031 (4)	−0.004 (4)

### Geometric parameters (Å, °)

**Table:**

Br—C1	1.920 (6)	C4—H4A	0.9300
O1—C2	1.213 (6)	C5—C6	1.387 (8)
C1—C2	1.502 (8)	C5—H5A	0.9300
C1—H1A	0.9700	C6—C7	1.400 (8)
C1—H1B	0.9700	C7—C8	1.363 (8)
O2—C6	1.359 (7)	C7—H7A	0.9300
O2—C9	1.412 (8)	C8—H8A	0.9300
C2—C3	1.474 (8)	C9—H9A	0.9600
C3—C8	1.380 (7)	C9—H9B	0.9600
C3—C4	1.393 (8)	C9—H9C	0.9600
C4—C5	1.370 (8)		
			
C2—C1—Br	113.3 (4)	C4—C5—H5A	119.4
C2—C1—H1A	108.9	C6—C5—H5A	119.4
Br—C1—H1A	108.9	O2—C6—C5	115.8 (5)
C2—C1—H1B	108.9	O2—C6—C7	125.6 (6)
Br—C1—H1B	108.9	C5—C6—C7	118.6 (6)
H1A—C1—H1B	107.7	C8—C7—C6	119.6 (6)
C6—O2—C9	118.7 (5)	C8—C7—H7A	120.2
O1—C2—C3	122.1 (6)	C6—C7—H7A	120.2
O1—C2—C1	120.8 (6)	C7—C8—C3	122.2 (6)
C3—C2—C1	117.0 (5)	C7—C8—H8A	118.9
C8—C3—C4	118.2 (6)	C3—C8—H8A	118.9
C8—C3—C2	118.3 (5)	O2—C9—H9A	109.5
C4—C3—C2	123.4 (6)	O2—C9—H9B	109.5
C5—C4—C3	120.3 (6)	H9A—C9—H9B	109.5
C5—C4—H4A	119.9	O2—C9—H9C	109.5
C3—C4—H4A	119.9	H9A—C9—H9C	109.5
C4—C5—C6	121.2 (6)	H9B—C9—H9C	109.5
			
Br—C1—C2—O1	0.0 (8)	C9—O2—C6—C5	172.0 (6)
Br—C1—C2—C3	−179.8 (4)	C9—O2—C6—C7	−6.3 (9)
O1—C2—C3—C8	2.2 (9)	C4—C5—C6—O2	−178.5 (6)
C1—C2—C3—C8	−178.0 (5)	C4—C5—C6—C7	0.0 (9)
O1—C2—C3—C4	−175.3 (6)	O2—C6—C7—C8	178.3 (6)
C1—C2—C3—C4	4.5 (9)	C5—C6—C7—C8	0.0 (9)
C8—C3—C4—C5	0.1 (9)	C6—C7—C8—C3	0.0 (9)
C2—C3—C4—C5	177.6 (6)	C4—C3—C8—C7	−0.1 (9)
C3—C4—C5—C6	0.0 (10)	C2—C3—C8—C7	−177.8 (6)

### Hydrogen-bond geometry (Å, °)

**Table:**

*D*—H···*A*	*D*—H	H···*A*	*D*···*A*	*D*—H···*A*
C8—H8A···O1	0.93	2.47	2.780 (8)	100
C7—H7A···O1^i^	0.93	2.58	3.505 (7)	171

Symmetry codes: (i) −*x*+1, *y*+1/2, −*z*+1/2.
